# Dietary Intervention Effectiveness, Clinical Outcomes and Nutrient and Salicylate Intakes in Older Adults Living in Long-Term Care Homes: The Results from the Senior’s Plate Project

**DOI:** 10.3390/nu14040871

**Published:** 2022-02-18

**Authors:** Danuta Gajewska, Paula Gosa, Paulina Katarzyna Kęszycka

**Affiliations:** Department of Dietetics, Institute of Human Nutrition Sciences, Warsaw University of Life Sciences (WULS), 159C Nowoursynowska Str, 02-776 Warsaw, Poland; pgosa129@gmail.com (P.G.); paulina_keszycka@sggw.edu.pl (P.K.K.)

**Keywords:** older adults, nursing home, nutrition, dietary intervention, dietary salicylates

## Abstract

Optimal nutrition is an important part of the therapeutic process offered to patients in long-term care, as it can significantly influence their nutritional and health status. The aim of this study was to assess the impacts of a dietary intervention on the nutritional status, clinical outcomes and selected nutrient and salicylate intakes among older adults living in a long-term care nursing home. To achieve the research goal, a prospective, non-randomized, baseline-controlled intervention study was conducted. The study was conducted within the framework of the “Senior’s Plate Project”, a project established in 2018 by the Polish Society of Dietetics. Methods: A 3 month dietary intervention, which included one serving of supplementary food, served as a second breakfast (Nestle Sinlac). Energy, nutrients and salicylates intakes were estimated on the basis of the menus. Food and beverage intakes among residents were verified by health care personnel. Anthropometric measurements and clinical examinations were conducted according to standard procedures at baseline and after intervention. Results: Of the 38 residents qualified for the study, 29 completed the program. Residents’ body mass index (BMI) values ranged from 13.3 kg/m^2^ to 34 kg/m^2^. A BMI < 22 kg/m^2^, indicating underweight, was found in 19 subjects. The dietary intervention resulted in increased body weight (57.8 ± 12.3 vs. 59.4 ± 12.6 kg), BMI (22.4 ± 4.0 vs. 23.0 ± 4.1 kg/m^2^) and body fat (19.2 ± 8.7 vs. 20.6 ± 8.9 kg). Significant changes in the levels of biochemical parameters, including serum calcium (8.7 vs. 9.5 mg/dL), potassium (4.1 ± 0.6 vs. 4.5 ± 0.5 mmol/L) and zinc (74.1 ± 10.9 vs. 109.0 ± 20.4 µg/dL), were observed. Energy, protein, fat and carbohydrate intakes were significantly higher in the third month of the intervention as compared to the baseline. The estimated medial daily intake of salicylates was low and ranged from 0.34 mg to 0.39 mg. Conclusions: The dietary intervention resulted in beneficial and significant changes in the nutritional status, biochemical parameters and nutrition of residents of the long-term care home. These results suggest that practical and individualized approaches are required to improve the nutritional status and clinical outcomes of nursing homes residents.

## 1. Introduction

According to a United Nations Report, one in every five people will be aged 60 years or over by 2050 [1], and the number of “oldest-old persons” (aged 80 years or over) will reach 20%. Demographic data indicate that the Polish society is one of the fastest ageing societies in Europe [2]. These older populations are considered significantly more nutritionally vulnerable than other population groups. One of the factors notably affecting the quality of life and health status of institutionalized older adults is appropriate nutrition. Research has revealed that several factors including physiological changes, decreased food intake, malabsorption, impaired sensory perception and lower physical activity may affect the body composition and nutritional status of older adults [3,4,5].

Optimal nutrition is an integral part of the therapeutic process offered to patients in long-term care [4,5,6,7,8]. High nutritional quality and optimal macro-, micro- and phytonutrient intakes are essential to ensure proper nutritional status and to prevent or delay the progression of age-related disease. It is unlikely that a single nutrient could significantly improve the health status or delay cognitive impairment in older age. Therefore, instead of a “one-size-fits-all” approach, a “whole-diet” approach and the combination of staple foods and super food or nutraceuticals is recommended as a potential strategy for healthy ageing [9]. This is particularly relevant in the nutrition of the institutionalized elderly, who are at high risk of malnutrition. The reported prevalence rates of protein energy malnutrition in the residential aged care setting ranged from 16% to 70%, depending on the tool used for assessment [10,11]. 

Dietary guidelines indicate that while energy requirements decrease with advanced age, the requirements for micronutrients remain the same as for younger adults. However, physiological changes, social changes and poor health status may place older adults at increased risk of micronutrient deficiency [12].

Among all food groups, fruits, vegetables, grains and legumes are important nutritional sources of vitamins, minerals, dietary fiber and diverse bioactive compounds, including polyphenols, carotenoids, plant sterols and salicylates. These natural bioactive constituents play an important role in the prevention and management of diseases, particularly age-related chronic diseases [13,14]. Greater consumption of fruits and vegetables by institutionalized elderly populations is associated with increased micronutrient intake [15,16]. However, the question remains whether diets rich in plant foods should be recommended as therapeutic nutritional interventions in older adults. 

We hypothesized that the nutritional intervention, tailored to residents’ needs, may improve their nutritional status and clinical outcomes. We also hypothesized that the diet of older adults is lacking in nutrients with anti-inflammatory properties, including salicylates. Therefore, we assumed that in planning a menu for this vulnerable group, and to increase the anti-inflammatory properties of the whole diet, we should be more mindful of food sources of dietary salicylates. Many common foods, such as fruits (berries, citrus fruits), vegetables, herbs, spices and beverages (black tea), contain salicylates and other bioactive components. However, the intake of these foods in older populations, for many reasons, is rather low. Furthermore, the consumption of herbs and spices is often over-looked in the studies on food intake. Hence, we strongly believe that it is important to assess the intake of foods with high concentrations of bioactive compounds (e.g., herbs and spices) that are consumed in small quantities. This is challenging, mainly due to methodological problems, including a lack of an international salicylate database and tools for accurate dietary salicylate evaluation. 

Considering the above, the aim of this study was to examine the effectiveness of a 3 month dietary intervention on nutritional status, biochemical parameters and nutrient and salicylate intake among older adults living in the nursing home. The study was undertaken within the framework of the Senior’s Plate Project, dedicated to institutionalized and community-dwelling older adults and their families and caregivers.

## 2. Materials and Methods

### 2.1. The Project

**Figure 1:**
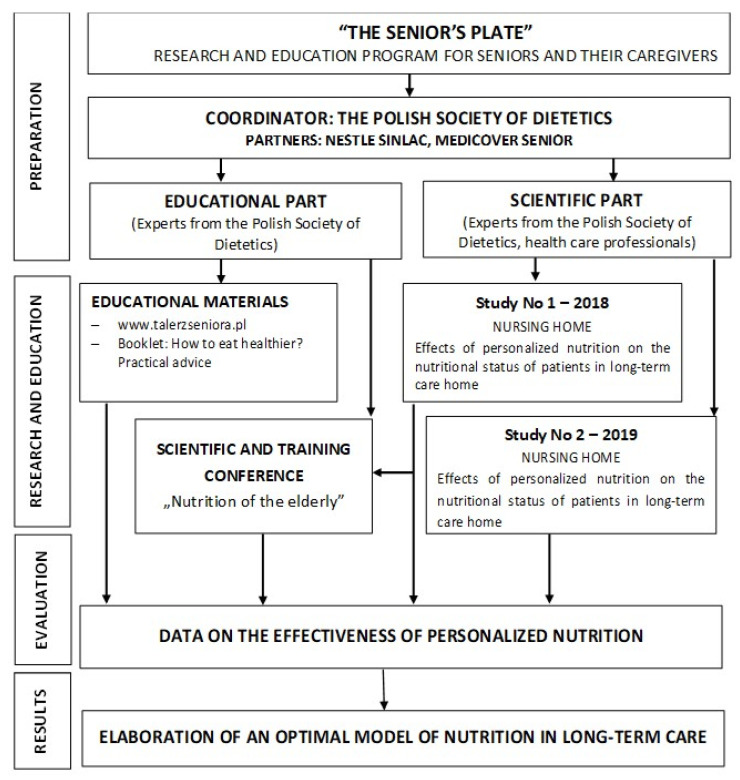
The Senior’s Plate Project design.

The study was conducted at a nursing home located near Warsaw (Poland), within the framework of the “Senior’s Plate” project. The project was launched in 2018 by the Polish Society of Dietetics. The program was addressed to seniors themselves and their caregivers (https://talerzseniora.pl, accessed on 20 December 2021). The main aim of the project was to evaluate the applicability and effectiveness of a personalized 3 month dietary intervention on the nutritional status and selected parameters of health of elderly individuals in long-term care (studies number 1 and number 2 in the scientific part of the project). The practical objective of the project was to formulate guidelines for an optimal model of nutrition for elderly individuals in long-term care in the context of the prevention of malnutrition (the educational part of the project). A detailed scheme of the project is shown in Figure 1. In this article, we present the results of study number 1. The results of study number 2 will be published elsewhere.

Research conducted under the project was composed of non-randomized studies of the effects of interventions (NRSI) and controlled before-and-after studies. The studies were conducted by dietitians from the Department of Dietetics of the Warsaw University of Life Sciences SGGW with the cooperation of nursing home health care professionals. Ethics approval for the study was obtained from the Ethics Committee of WULS-SGGW (No 7/2017). All procedures were conducted according to the international ethical principles of the Declaration of Helsinki. Studies were conducted between June 2018 and September 2019. 

### 2.2. Study Participants

The selection of patients for the study was purposeful and voluntary. The study population consisted of 38 residents, remaining in long-term residential care for 1 to 3 years. Adults of both sexes who provided written informed consent were included in the study. The consent for participation of seniors in the study was also obtained from their caregivers, who were provided with comprehensive information about the study (course, purpose and scope). The study did not include seniors with psychiatric disorders, subjects on enteral or parenteral nutrition, and subjects who did not give written consent to participate in the study. Of the 38 residents eligible for study number 1, 29 seniors completed the interventional study, including 7 men and 22 women. Reasons for not completing the program were patient deaths, transfer to another nursing home, or health status that prevented participation. 

The nursing home provided home care, day care and long-term residential care for a diverse group of older adults, including those with dementia, Alzheimer’s disease, Parkinson’s disease and stroke. All individuals are admitted to this nursing home, regardless of their health status or level of dependency.

### 2.3. Anthropometric and Body Composition Measurements

Anthropometric measurements, including body weight (kg) and height (cm), were taken in the morning by the personnel caring for the patients at baseline and at 3 months follow-up as part of a routinely monitored nutritional status. The BMI was calculated from the patients’ weight and height. If standing height was unable to be obtained, estimated current height was used to calculate the BMI. The interpretation of the BMI was based on the guidelines for the elderly [17].

Body composition was measured by trained staff using multiple-frequency bioelectrical impedance analysis (BIA) according to the standard procedures [18]. Resistance (R) and reactance (Xc) were measured using a four-terminal impedance analyzer (model BIA-101, RJL system, Detroit). Values obtained from bioimpedance measurements were related to reference values for older adults [19]. Due to health status or other factors interfering with the reliability of the results, the measurements were performed among 29 residents at the baseline and among 26 at the follow-up. 

Fat-Free Mass Index (FFMI) was calculated from the formula: FFMI (kg/m^2^) = FFM_BIA_ (kg)/(height)^2^ (m). FFMI < 15 (F) kg/m^2^ or < 17 (M) kg/m^2^ was interpreted as low [19,20]. The fat mass index (FMI) was calculated from the formula: FMI (kg/m^2^) = FMBIA (kg)/(height)^2^ (m). Body fat (%) was calculated from the formula: BF% = FM (kg)/body mass (kg) × 100 [20,21]. 

### 2.4. Laboratory Tests

Baseline and follow-up biochemical tests were conducted with the consent of the geriatrician in an accredited laboratory using standard analytical methods. Blood for determinations was collected from residents in the morning in fasting state. Blood samples were transported to the laboratory on ice. Serum samples were separated by centrifugation, according to laboratory standard procedures, within 0.5–1 h following collection. The analyses of TSH, folic acid and vitamin B12 were performed using an electrochemiluminescence immunoassay (ECLIA). C-reactive protein (CRP) was measured using the immunoturbimetry method. The potassium level was measured using indirect potentiometry. The zinc concentration was determined using flame atomic absorption spectrophotometry. Hemoglobin, total cholesterol, HDL-cholesterol and LDL-cholesterol fractions, triglycerides, glucose, uric acid, iron and calcium were determined using spectrophotometric methods. Analyses were performed using the automatic blood biochemical analyzer Cobas 6000/e 601 module (Roche Diagnostic, Switzerland). The results were compared with laboratory standards.

### 2.5. Dietary Intervention

All participants received a regular diet, or when essential therapeutic diets (diabetic, with modified texture). All meals were prepared in the nursing home kitchen. Weekly menus were posted in a common area for residents and their families to see. The menus included three meals (breakfast, lunch and supper) and two snacks per day. Standardized recipes were used to prepare all foods and beverages. Menus were revised by a dietitian and adjusted to the seasonality of foods and resident’s liked or disliked food. Additionally, during a 3 month period of the dietary intervention, all residents were given one serving of supplemental food, served as a second breakfast. The cereal product Nestlé Sinlac was selected as the product for supplementation. One serving of this product provided 212 kcal, 5 g of fat, 34.4 g of carbohydrates, 6.75 g of protein, 1.4 g of fiber and various vitamins (A (205 µg), D (2.25 µg), E (1.25 mg α-TE), C (32.5 mg) B_1_ (0.45 mg), B_2_ (0.17 mg), B_6_ (0.13 mg), B_12_ (0.4 mg), niacin (1.5 mg), pantothenic acid 0.55 µg), biotin (10 µg)) and minerals (calcium (265 mg), iron (4 mg), zinc (2.8 mg), iodine (27.5 µg), sodium (57.5 mg)). The choice of this supplement was based on it being a gluten-free and lactose-free product containing no strong allergens (cow milk protein, soy) that is easy to prepare (by seniors themselves or the staff of the nursing home), neutral in taste, contains probiotics (*Bifidobacterium lactis*), is available on the market and has received positive opinions and reviews from dietitians in the care of elderly patients. There were no side effects of supplementation.

Individual goals regarding energy requirements, dietary intake and body weight were defined by an interdisciplinary team before the study. Individual energy requirements were calculated based on an empirical equation, taking into account the age, sex, weight and height of the patients [22], as well as their activities. According to the Polish Dietary Guidelines, the recommended proportions of energy from protein, fats and carbohydrates in the diet were 15–20%, 20–35% and 45–60% of the total energy, respectively [23]. The macronutrient composition of the nursing home menus provided during the 3 month dietary intervention is presented in Table 1.

The dietary intervention was monitored and evaluated for effectiveness. Diet adherence was assessed before the intervention and during the last month of the intervention. A total of 21 menus were taken into account in the calculations, 7 from each month of intervention. As some residents did not consume all of the foods and beverages that were included in the menus, the amount of food intake was verified by health care personnel observation during meal consumption using a clinician-reported outcome assessment method. An individual resident’s food intake was scored as the percentage of each serving actually consumed and coded as follows: 1—whole portion; 2—3/4 portion; 3—1/2 portion; 4—1/4 portion; 0—no intake. The energy and nutrient analysis of the menus was performed using Dieta 5.D software (National Food and Nutrition Institute, Warsaw, Poland). 

### 2.6. Assessment of Food Intake and Intake

The food and beverage intakes of the elderly were assessed based on the menus prepared for the residents of the nursing home. The assessment was based on weekly menus.

The total salicylate intake was calculated on the basis of up-to-date databases on salicylate contents in food [24,25,26].

### 2.7. Statistical Analysis

All data were collected and entered into an Excel database (Microsoft Corp., Redmont, WA, USA). The normality of data distribution was verified using the Shapiro–Wilk test. Continuous data are presented as means and standard deviations (SDs) or medians and categorical data as numbers or percentages (%). The descriptive characteristics of the participants are presented by quartiles. The Wilcoxon signed-rank test and Student’s *t*-test were used to assess baseline and follow-up results for non-normally distributed and normally distributed data, respectively. Pearson correlation coefficients were used to assess the relationships between age and body composition parameters. Data were analyzed using Statistica 14 (StatSoft Inc., Tulsa, OK, USA). A statistical significance level of *p* < 0.05 was adopted for all analyses. 

## 3. Results

### 3.1. Study Participants

Table 2 presents baseline characteristics of the study population. Thirty-eight subjects were involved in the study, including 29 women (76.3%) and 9 men (23.7%). The youngest resident in long-term care was 71 years old, while the oldest was 99 years old. The largest group (20 individuals) consisted of residents aged 81-90 years, followed by residents aged over 90 years (10 individuals) and 71–80 years (8 individuals). 

The residents’ BMI values ranged from 13.3 kg/m^2^ to 34 kg/m^2^. BMI values below 22 kg/m^2^, indicating unacceptably low body weight, were found in 19 residents. BMI values above 27 kg/m^2^, indicating unacceptably high body weight, were found only in the female group (Table 3). The BMI values of the residents were significantly related to age (*p* = 0.016, r^2^ = 0.155). The mean body fat (FM) did not exceed 20 kg, which with respect to reference values, should be considered low. The phase angle (PA), FM and FFM values of residents were negatively, not significantly, correlated with age (*p* = 0.064, r^2^ = 0.121; *p* = 0.152, r^2^ = 0.074; *p* = 0.174; r^2^ = 0.067, respectively).

Before the dietary intervention, only 13 participants in the study were able to consume meals independently. The remaining residents were dependent on others for eating. Health care personnel observations prior to the dietary intervention indicated that only 10 seniors consumed the entire meal served, and 1/2 or 1/4 of the portion was consumed by as many as 14 seniors. After the nutritional intervention, the patients’ appetites, measured using a clinician-reported outcome assessment method, improved and the total food intake increased. The number of patients consuming the entire serving increased to 21.

### 3.2. Health Status of Participants 

The health status of participants is demonstrated in Table 4. Multimorbidity, defined as the co-occurrence of two or more diseases or conditions in the same individual, was found in all study participants. The most commonly diagnosed diseases were hypertension (16 residents) and Alzheimer’s disease (13 residents) (Table 3). Eight individuals were diagnosed with two diseases, 8 individuals with three diseases, 7 individuals with four diseases, 3 individuals with six conditions and 1 individual with as many as eight diseases and conditions. 

### 3.3. Changes in Nutritional Status of Participants after Dietary Intervention 

After completion of the 3 month dietary intervention, statistically significant increases in the participants’ body weight, BMI, fat mass and FMI were observed. The mean BMI increased from 22.4 ± 4.0 to 23.1 ± 4.1 kg/m^2^. The minimum BMI increased from 13.3 to 14.8 kg/m^2^. Three patients experienced a decrease in BMI, including one overweight resident. The other changes in measured parameters were not statistically significant (Table 5). 

**Figure 2:**
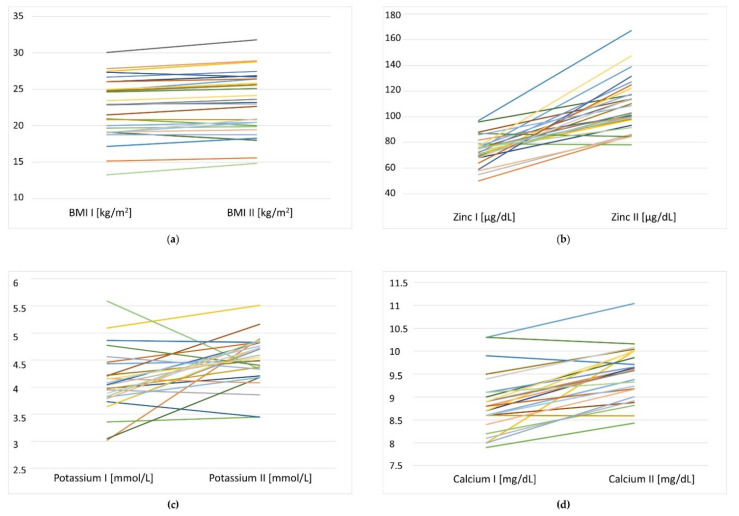
Individual changes in (**a**) BMI (*n* = 29), (**b**) serum zinc (*n* = 29), (**c**) serum potassium (*n* = 28) and (**d**) serum calcium (*n* = 26) in the study participants at baseline (I) and after dietary intervention (II).

The assessment of the hematological and clinical chemistry results before and after the dietary intervention showed significant changes in parameters such as fasting glucose, total cholesterol, high-density (HDL) cholesterol and low-density (LDL) cholesterol, as well as serum zinc, potassium and calcium (Table 6). Individual statistically significant changes in BMI, serum zinc, potassium and calcium in the study participants at baseline and after dietary intervention are presented in Figure 2. 

### 3.4. Changes in Energy and Nutrient Intakes after Dietary Intervention 

Energy and macronutrient intakes at baseline and after the 3 month dietary intervention are presented in Table 7. Before the dietary intervention, only 13 participants in the study were able to consume meals independently. The remaining elderly participants required assistance from staff during meal consumption. The dietary intervention resulted in significant improvements in energy, protein, carbohydrate and fiber intakes, as well as increases in saturated fat and cholesterol intakes. After the dietary intervention, we also observed significant increases in intakes of all micronutrients as compared to baseline, except vitamins E and C (Table 8). 

### 3.5. Salicylate Content in Older Adults’ Diet

Table 9 and Table 10 show the contents of salicylates in the nursing home resident’s diet, estimated for each month of the study intervention, as well as the main food sources of salicylates. The estimated medial daily intake of salicylates was low and ranged from 0.34 mg to 0.39 mg. The main sources of dietary salicylates were vegetables (64%) and cereals (31%). Fruits provided only 2% of the total daily salicylates. Likewise, herbs and spices contributed 2% of the total amount of salicylates. 

## 4. Discussion

In this study, we present the data regarding the impacts of a unimodal 3 month dietary intervention on the nutritional status, macronutrient, micronutrient and salicylate intakes among individuals aged 70 years or older living in a nursing home. Over 60% of the study population was over 85 years of age. We found this group of institutionalized older patients to be highly heterogenous with respect to their health and nutritional status, as well as their needs for assistance in activities of daily living. Half of the nursing home residents were affected by malnutrition. The majority of them experienced concurrent diseases, and in most cases the diseases were inflammatory in nature. 

The baseline examination of the nutrition of study participants revealed an inadequate energy intake, as well as low folate, potassium, calcium and magnesium. During the 3 month nutritional intervention, all individuals received oral food supplementation (once a day) in the form of a lactose-free and gluten free-cereal porridge. 

A multimodal approach, including dietary and exercise interventions, is recommended to improve muscle mass, muscle strength and physical performance and to prevent functional decline in older adults [27]. However, regarding health status, nutritional status and limitations of mobility in older individuals, the applicability of the multimodal approach is limited. According to ESPEN guidelines (2018), nutritional interventions for older individuals should be part of a multimodal and multidisciplinary team intervention in order to support adequate dietary intake, maintain or increase body weight and improve functional and clinical outcomes [4]. Our study was a unimodal nutritional intervention. Individual goals regarding energy requirements, amounts of food and body weight status were defined on the basis of anthropometric measurements and the health status of older persons. The recommended diets and food serving sizes were tailored to patient needs and preferences. This simple dietary intervention resulted in significant improvements in food intake, body mass and selected biochemical parameters. Some changes, such as increased fasting glucose, total cholesterol and LDL cholesterol, were rather negative. It is, therefore, difficult to assess whether these changes were related to the nutritional intervention or were associated with an underlying disease. 

The dietary intervention resulted in increased intake of energy, protein, carbohydrates and fiber, as well as saturated fats and cholesterol. Higher intakes of SFA and cholesterol have been shown to be associated with increased inflammation and higher risk factors for cardiovascular diseases and metabolic disorders. In general, a healthy diet that is rich in high-quality and nutrient-dense foods is recommended at all stages of the life span. This is particularly important in later life, when age-related diseases are more prevalent. However, in the nutrition of older adults, a more liberal diet tailored to the person’s needs should be considered. Nutrition and a palatable diet are essential components of older adults’ quality of life. Dietary restrictions may limit food intake and increase the risk of malnutrition among older persons. Older adults who reside in long-term homes are particularly vulnerable to malnutrition and their nutritional requirements are not well defined. Therefore, the benefits and risks associated with dietary restrictions should be carefully considered by the health care team. Although therapeutic diets are required to treat diseases, they may result in limited food choices, a decline in appetite, poor food intake and undernutrition. 

As a result of the nutritional intervention, we found increased appetite, food intake and nutrient intake among residents. This resulted in weight gain, a higher body mass index and significant increases in fat mass for the residents. Food intake typically declines with age, even in healthy older adults. Changes in appetite could be associated with age-related physiological changes, as well as changes in hormones levels that control food intake and satiety. Older adults with small appetites require the implementation of several strategies to provide sufficient energy and nutrients. 

One of the beneficial changes observed after nutritional intervention was the significant improvement of the serum zinc status of residents (74.1 ± 10.9 vs. 109.0 ± 20.4 µg/dL, *p* = 0.000). Zinc deficiency is a global problem. Studies have proven that the fortification of staple foods with zinc may be an effective strategy for preventing zinc deficiency. However, fortification was effective if zinc was the only micronutrient used for fortification (not in combination with other nutrients) [28]. Supplementation with cereal products provided an additional 2.8 mg of zinc daily in the diet of residents. It can be hypothesized that this had the effect of reducing age-related anorexia and improving appetite. However, this problem needs to be studied in more detail.

There is no specific micronutrient recommendation for older persons and the recommendation does not differ significantly from the one for younger adults. Micronutrient malabsorption is not a consequence of ageing. However, many older adults suffer from diseases (atrophic gastritis, hypochlorhydria), which leads to reductions in nutrient bioavailability and increases the risk of micronutrient deficiency. Prolonged suboptimal intake of micronutrients may lead to a decline in cognitive performance. Very old individuals may have different nutritional requirements for energy and nutrients as compared to younger individuals. Therefore, the World Health Organization (WHO) has called to review the existing nutritional guidelines for this vulnerable and growing population. The WHO emphasizes that aging and nutrition are growing global challenges [12].

There are many types of dietary approaches that can be used to prevent or treat malnutrition in older adults, including fortifying normal food and beverages, modifying meal profiles (patterns, textures), increasing the frequency of meals, providing additional snacks or meals, providing advice to eat high-protein, high-energy foods and the use of commercially available oral nutritional supplements (ONS). Most of these strategies are cost-effective [4,5,6,7,29,30]. The systematic literature review by Hugo et al. [7] provides evidence that nutritional interventions (supplements and food-based nutrition) may be cost-effective in improving clinical outcomes associated with malnutrition in age care settings. Most interventions aimed to provide supplementation of 300–400 kcal and 12–20 g of protein per serving, as well as additional vitamins and minerals [6].

Adequate intakes of dietary proteins from animal and plant sources and the timing of protein intake are important to prevent the loss of muscle mass and physical decline in older adults. Protein intake among residents in our study population was higher than the current European Recommended Dietary Allowance (RDA) for older adults, as indicated by the European Food Safety Authority [31]. According to ESPEN guidelines, the daily protein intake in older individuals should be at least 1 g protein per kg of body weight and should be individually adjusted with regard to nutritional status, physical activity level, disease status and tolerance [4]. However, some experts have suggested that older adults have a greater requirement for protein than RDA. They propose higher protein intakes (up to 1.5 g/kg body weight/day) to reduce progressive loss of muscle mass in older adults [32,33,34]. In addition to the adequate amount of protein in the diet of older adults, the distribution of protein across meals during the day could be an efficient strategy to optimize muscle protein synthesis. A diet pattern containing between 25 and 30 g of protein at each meal has been proposed as the most efficient approach [35,36]. 

One important element of the dietary assessment in the present study was to evaluate the intake of salicylates and their dietary sources. Our finding revealed that the main sources of dietary salicylates were plant foods, including vegetables and cereals. However, the intake of salicylates was very low at 0.45 mg/day. This value differed significantly from the other authors’ findings, who estimated daily salicylate intake to range from 3 mg to 200 mg [37,38]. Interestingly, in our previous study, we found that vegan diets provided a higher amount of dietary salicylates from only herbs and spices, as compared to the entire seniors’ menus [39].

These phytonutrients naturally occur in many food products and display pharmacological properties in the prevention of cardiovascular diseases, several types of cancer and diabetes [40,41,42,43]. However, intakes of those compounds are not routinely assessed in populations. To the best of our knowledge, diets of nursing home residents were not analyzed for dietary salicylate content. In our opinion, dietary salicylates may be potential therapeutic agents; therefore, food rich in salicylates should be recommended for older adults residing in health care communities. Furthermore, dietary salicylates and other anti-inflammatory compounds (from food and beverages) should be taken into consideration when planning intervention strategies for the prevention or management of age-related diseases. This strategy (high-salicylate diet) seems to be more safe and more beneficial than drug prevention or treatment (aspirin). Some studies have highlighted that aspirin use in older adults (in low dose) may not reduce the risk of cardiovascular disease more than a placebo [44,45]. 

The hypothesis that dietary salicylates have contributed to the decline in mortality attributable to cardiovascular diseases (salicylate–cardiovascular disease hypothesis) was formulated in 1997 by Ingster and Feinleib [46]. Further research is still needed to confirm this hypothesis. Further studies confirmed that the intake of food rich in salicylates (fruits, vegetables, herbs and spices) increased the concentration of salicylic acid in humans [47,48,49]. Although many factors may influence the concentration of salicylic acid and its derivatives in food, as well as the bioavailability of these compounds in humans, further research is still needed to formulate the recommendations related to salicylate intake. However, we emphasize that good sources of salicylates should be considered in planning and evaluating the nutritional value of diets of the elderly.

### Strengths and Limitations of the Study

The strengths of the study include the intervention strategy and the involvement a multidisciplinary team with a dietitian. The individualized care plans used for all residents and the unimodal approach make the model of the study appropriate for documenting the impacts of dietary interventions on the health status and nutritional status of study participants. 

However, certain study limitations must be considered. The sample size was relatively small and the group of participants was relatively heterogeneous; therefore, our findings may not be generalized. The trial was not blinded and the follow-up periods of 3 months might be too short for observations and long-term results. The study was not randomized and the involvement of a control group was not feasible. We did not measure the quality of life of residents and assessments of muscle strength were not included in the assessment of nutritional status.

## 5. Conclusions

The dietary intervention may result in significant improvements in clinical outcomes in older adults. Diets that are tailored to older individual’s needs, desires and medical conditions may help to meet nutritional recommendations and improve the nutritional status of older adults. This seems to be a safe, effective and beneficial strategy to prevent malnutrition in institutionalized older adults. Nevertheless, more research on the role of personalized nutrition and malnutrition prevention strategies in older adults is still needed.

## Figures and Tables

**Table 1:** Total energy and macronutrient distribution of the nursing home menus.

Macronutrient	First Month of the Study	Second Month of the Study	Third Month of the Study	An Average for 3 Month Intervention	Recommended Value ^1^
Total energy, kcal	1851 ± 136	1958 ± 226	1832 ± 198	1870 ± 154	1600–2600 ^2^
Protein, %	17.9 ± 2.2	16.8 ± 2.7	17.5 ± 1.8	17.4 ± 2.2	15–20
Fat, %	31.0 ± 5.5	31.1 ± 4.3	29.1 ± 4.9	30.4 ± 4.8	20–35
Carbohydrates, %	51.1 ± 4.5	52.1 ± 4.0	53.4 ± 5.0	52.2 ± 4.7	45–65

Values are means ± standard deviation; ^1^ according to Jarosz et al. [23]; ^2^ individualized recommendations.

**Table 2:** Baseline characteristics of the study population (*n* = 38).

Parameters	Mean	SD	Minimum	Median	Maximum
Age, years	86.3	7.2	71	86	99
Weight, kg	57.4	12.4	30.0	58.0	78.0
BMI, kg/m^2^	21.9	3.9	13.3	22.2	34.0
^1^ FM, kg	19.2	8.3	2.4	20.5	36.0
^1^ FFM, kg	40.9	6.5	29.5	41.3	57.0
^1^ FFMI, kg/m^2^	15.7	1.8	12.6	15.7	19.3
^1^ PA, °	4.0	0.7	2.9	4.1	5.3

SD—Standard Deviation; BMI—Body Mass Index; FM—Fat Mass; FFM—Fat-Free Mass; FFMI—Fat-Free Mass Index; PA—Phase Angle; ^1^ *n* = 29.

**Table 3:** Baseline body weight status of seniors.

Body Weight Categories *	Male (*n* = 9)	Female (*n* = 29)
Underweight (BMI < 22 kg/m^2^)	5	14
Normal (BMI 22–27 kg/m^2^)	4	11
Overweigh and obesity (BMI > 27 kg/m^2^)	0	4

BMI—Body Mass Index; * according to Lipschitz [17].

**Table 4:** Group of diseases diagnosed among study participants.

Diseases	Number of Patients (*n* = 38)	Percentage (%)
Hypertension	16	42.1
Alzheimer’s disease	13	34.2
Dementia ^1^	10	26.3
Hypothyroidism	5	13.2
Cardiovascular diseases, CVD ^2^	8	21.0
Type 2 diabetes	7	18.4
Osteoarthritis	4	10.5
Dyslipidemias	5	13.2
Parkinson’s disease	3	7.8
Chronic kidney disease	2	5.2

^1^ Mixed dementia, post stroke, vascular; ^2^ CVD—cardiovascular diseases (heart failure, stroke, ischemic heart disease, heart rhythm disorders).

**Table 5:** Changes in body mass and body composition among elderly individuals at baseline and after dietary intervention.

Parameters	Baseline	After Dietary Intervention	*p*-Value
Body mass (kg) (29)	57.8 ± 12.3	59.4 ± 12.6	0.000
BMI (kg/m^2^) (29)	22.4 ± 4.0	23.1 ± 4.1	0.000
FM (kg) (26)	19.2 ± 8.7	20.6 ± 8.9	0.006
FMI (kg/m^2^) (26)	7.4 ± 3.3	7.9 ± 3.4	0.006
FFM (kg) (26)	40.9 ± 6.3	40.9 ± 5.9	0.864
FFMI (kg/m^2^) (26)	15.8 ± 1.8	15.8 ± 1.7	0.976
BF (%) (26)	31.4 ± 10.8	32.6 ± 10.0	0.131
PA (°) (26)	4.1 ± 0.7	4.3 ± 1.0	0.153
TBW (%) (26)	32.7 ± 5.1	32.6 ± 4.7	0.813
ECW (%) (26)	18.6 ± 2.3	18.1 ± 2.3	0.160

(*n*)—Number of patients; *p*-value, Student’s test; BMI—Body Mass Index; FM—Fat Mass; FMI—Fat Mass Index; FFM—Fat-Free Mass; FFMI—Fat-Free Mass Index; BF—Body Fat; PA—Phase Angle; TBW—Total Body Water; ECW—Extracellular Water.

**Table 6:** Comparison of the hematological and clinical chemistry results before and after dietary intervention.

Parameters	Baseline	After Dietary Intervention	*p*
Hemoglobin (g/dL) (28)	12.1 ± 1.8	12.0 ± 2.2	0.947 *
Hematocrit (%) (28)	37.7 ± 5.7	35.9 ± 7.7	0.222 *
Platelet Count (10^9^/L) (28)	224 ± 75	216 ± 59	0.423 *
White Blood Cell (10^9^/L) (28)	6.2	5.6	0.456 **
Fasting glucose (mg/dL) (27)	73.0	76.4	0.009 **
Uric acid (mg/dL) (26)	5.0 ± 1.9	5.1 ± 1.7	0.829 *
Total Cholesterol (mg/dL) (26)	183 ± 41	200 ± 45	0.001 *
HDL-Cholesterol (mg/dL) (26)	57.5 ± 14	61.9 ± 17	0.015 *
LDL-Cholesterol (mg/dL) (26)	105.4 ± 32	119.2 ± 34	0.000 *
Triglycerides (mg/dL) (26)	87.8	101.3	0.675 **
TSH (µIU/mL) ((25)	1.69	2.01	0.637 *
CRP (mg/dL) (27)	0.17	0.252	0.186 **
Folic acid (ng/mL) (25)	8.3 ± 6.1	7.4 ± 3.6	0.208 *
Vitamin B_12_ (pg/dL) (27)	301.0	312.3	0.102 **
Zinc (µg/dL) (29)	74.1 ± 10.9	109.0 ± 20.4	0.000 *
Potassium (mmol/L) (28)	4.1 ± 0.6	4.5 ± 0.5	0.002 *
Calcium (mg/dL) (26)	8.7	9.5	0.000 **
Iron (µg/dL) (27)	72.6 ± 24.6	79.4 ± 22.7	0.168 *

Values are medians or means ± standard deviations; (*n*)—number of patients; *p* *—Student’s test; *p* **—Wilcoxon signed-rank test.

**Table 7:** Energy and macronutrient intakes at baseline and after the dietary intervention among study participants (*n* = 29).

Parameters	Baseline	Last Week of Intervention ^1^	*p*-Value
Energy (kcal/day)	1504	1912	0.034
Protein (g/day)	61.9	82.3	0.006
Protein (g/kg bw/day)	1.02	1.18	0.014
Fat (g/day)	51.8	62.7	0.705
Fat (g/kg bw/day)	0.85	0.93	0.721
SFA (g/day)	19.2	24.3	0.006
MUFA (g/day)	19.1	21.6	0.974
PUFA (g/day)	6.83	7.71	0.991
Cholesterol (mg/day)	247	231	0.022
Carbohydrates (g/day)	206	273	0.006
Carbohydrates (g/kg bw/day)	3.16	3.74	0.015
Saccharose (g/day)	7.9	12.7	0.000
Dietary fiber (g/day)	19.2	24.2	0.007
Dietary fiber (g/1000 kcal)	13.6	15.1	0.000

Note: *p* value, Wilcoxon signed-rank test; bw/d—body weight/day; ^1^ including energy and nutrients from supplement.

**Table 8:** Micronutrient intake at baseline and after dietary intervention among study participants (*n* = 29).

Parameters	Baseline	Last Week of Intervention ^1^	*p*-Value
Sodium (mg/day)	1961 ± 658	2430 ± 401	0.006
Potassium (mg/day)	2134 ± 758	2732 ± 483	0.000
Calcium (mg/day)	667 ± 211	760 ± 160	0.007
Phosphorus (mg/day)	913 ± 329	1130 ± 221	0.006
Magnesium (mg/day)	212 ± 77	281 ± 58	0.000
Iron (mg/day)	12.4 ± 3.9	14.8 ± 2.9	0.000
Zinc (mg/day)	10.2 ± 3.3	12.1 ± 2.6	0.006
Cooper (mg/day)	0.88 ± 0.3	1.1 ± 0.2	0.000
Iodine (µg/day)	66.0 ± 20.8	72.8 ± 15.7	0.006
Vitamin A (µg RE/day)	1419 ± 477	1759 ± 304	0.002
Vitamin E (mg α-TE)/day)	8.8 ± 2.9	8.8 ± 1.5	0.974
Thiamin (mg/day)	1.6 ± 0.5	1.9 ± 0.3	0.000
Riboflavin (mg/day)	1.2 ± 0,4	1.4 ± 0.3	0.020
Niacin (mg/day)	13.6 ± 4.5	17.4 ± 2.9	0.006
Pyridoxin (mg/day)	1.7 ± 0.5	2.0 ± 0.3	0.000
Folate (µg/day)	267.7 ± 99	334.6 ± 55	0.039
Cobalamin (µg/day)	2.3 ± 0.3	2.8 ± 0.4	0.000
Vitamin C (mg/day)	130.7 ± 42	138.6 ± 26	0.405

Note: *p* value, Wilcoxon signed-rank test; ^1^ including energy and nutrients from supplement; RE—retinol equivalents; α-TE—tocopherol equivalents.

**Table 9:** Salicylate content in the nursing home menus (mg/day).

	First Month of the Study	Second Month of the Study	Third Month of the Study	An Average for 3 Month Intervention
Average	0.41	0.45	0.39	0.42
SD	0.23	0.22	0.25	0.22
Minimum	0.15	0.30	0.17	0.15
Median	0.39	0.37	0.34	0.37
Maximum	0.68	0.94	0.91	0.94

SD—Standard deviation.

**Table 10:** Estimated amount of dietary salicylates in seniors’ diets from food groups (mg/day).

	Fruits	Vegetables	Cereals	Meat and Meat Products	Meat Alternatives	Spices
Average	0.01	0.27	0.13	0.003	0.002	0.01
SD	0.03	0.14	0.21	0.001	0.003	0.01
Minimum	0	0.02	0.02	0	0	0
Median	0	0.27	0.05	0.003	0	0.01
Maximum	0.10	0.58	0.74	0.006	0.012	0.02

SD—Standard deviation.

## Data Availability

The data presented in this study might be available on request from the corresponding author.
